# Comparison of the antiangiogenic activity of modified *RGDRGD*-endostatin to endostatin delivered by gene transfer in vivo rabbit neovascularization model

**Published:** 2011-07-15

**Authors:** Hong-yan Ge, Nan Xiao, Xiu-li Yin, Song-bin Fu, Jin-ying Ge, Yan Shi, Ping Liu

**Affiliations:** 1 Key Laboratory of Harbin Medical University Eye Center, Eye Hospital, First Affiliated Hospital, Harbin Medical University, Harbin, P.R. China; 2 Laboratory of Medical Genetics, Harbin Medical University, Harbin, P.R. China; 3 Chinese Academy of Agriculture Sciences, Harbin Veterinary Research Institute, Harbin, P.R. China

## Abstract

**Purpose:**

Endostatin plays an important role in inhibiting corneal neovascularization (CNV). The aim of this study was to evaluate the antiangiogenic activities of lipid-mediated subconjunctival injection of the modified RGDRGD (arginine- glycin- aspartic- arginine- glycin- aspartic- endostatin gene in a rabbit model of neovascularization in vivo.

**Methods:**

A modified human endostatin gene containing an *RGDRGD* motif was obtained by rapid site-directed mutagenesis. Forty New Zealand white rabbits underwent alkaline burn and developed CNV, which were randomly divided into four groups: an experimental control group, a PCI empty vector group, a PCI-endostatin group, and a PCI-*RGDRGD*-endostatin group. The vector, endostatin, and *RGDRGD*-endostatin groups received injections into the superior bulbar conjunctiva after the burn. An injection of 5 μg was given twice at 1-week intervals. Four eyes of two rabbits received neither treatment nor alkaline burn and served as absolute normal controls. The areas of CNV were monitored after 7 and 14 days. Corneas were examined by histology, and VEGF (vascular endothelial growth factor) and CD31 (platelet endothelial cell adhesion molecule-1) expression was detected by immunohistochemistry after 7 and 14 days. Retina, liver, and kidney were examined by histology, and CD38 expression in the inflammatory cells was detected by immunohistochemistry at 90 days.

**Results:**

Subconjunctival injection of both native endostatin and modified *RGDRGD*-endostatin genes resulted in a significant suppression of CNV in vivo, with modified *RGDRGD*-endostatin being more effective than native endostatin. The mean concentration of VEGF in the PCI-*RGDRGD*-endostatin group significantly decreased compared to the means in the other groups. Upon histological examination, the endostatin-treated and *RGDRGD*-endostatin-treated eyes showed significantly less neovascular area and fewer vessels than the control and vector-injected groups. Retinal, hepatic, and renal tissue sections were normal, and there was no inflammatory cell infiltration observed.

**Conclusions:**

Native and modified endostatin can significantly inhibit CNV by suppressing the expression of VEGF. However, modified endostatin with the *RGDRGD* motif is far more effective than the endostatin gene in antiangiogenic activity.

## Introduction

Angiogenesis or neovascularization, the development of new capillaries from preexisting blood vessels, is an important process in pathophysiologic situations. Corneal neovascularization (CNV) occurs when the balance between angiogenic and antiangiogenic factors is tilted toward angiogenic factors; this imbalance can lead to corneal scarring, edema, lipidic deposition, and inflammation, resulting in significant visual impairment [1,2]. CNV is a severe complication induced by various pathogenetic factors: it is one of the most common causes of visual impairment and it is a high-risk factor for rejection after allograft corneal transplantation [3-6]. CNV can be a profoundly debilitating condition, leading to loss of the immunologic privilege of the cornea and severe visual impairment [7].

Clinically, CNV is one of the most difficult diseases to treat. Several angiogenesis inhibitors have been identified and tried as medical treatments, including prolactin [1,8], angiostatin [9], cyclosporine A [3], TNP-470 {[O-[chloroacetyl-carbamoyl]fumagillol, initially called AGM-1470]} [10], steroids [11], endostatin [12], and suramab [13]. Studies of Hamid et al. [14] showed that the application of anti–vascular endothelial growth factor (VEGF) agents inhibited CNV. Felix et al. [15] and Erdurmus et al. [16] found that bevacizumab potently inhibited inflammatory corneal angiogenesis. Sharma et al. [17],  found that angiotensin converting enzyme (ACE) inhibitors might represent a novel therapeutic strategy to treat corneal angiogenesis. Lai’s studies [18] suggested that the expression of adenovirus-mediated sFlt-1 inhibited the angiogenesis in a rat model of CNV. Recently, gene therapy was the focus in CNV. Yoon et al. [1] reported that lipid-mediated delivery of brain-specific angiogenesis-inhibitor 1 gene reduces rabbits’ CNV. Murthey et al. [19] demonstrated that gene transfer of kringle 5 of plasminogen inhibited CNV. Zuo et al. [20] found that synthetic small interference RNA (siRNA), targeting VEGF-A, could inhibit mouse CNV. Wu et al. [21] found potential feasibility in the local application of vasostatin (VS) or treatment of corneal angiogenesis. Peng et al. [22] indicated that gene therapy with the recombinant, retroviral, vector-hosted mEndo, and msFlk-1 genes effectively inhibited CNV induced by alkaline burn. So far, however, there is no ideal treatment method for CNV. For this reason, it is very important to find an effective method to treat CNV.

Endostatin is a 22,000 molecular weight (M_r_) COOH-terminal fragment of collagen XVIII, a component of the basement membrane, that specifically inhibits VEGF and bFGF-induced endothelial proliferation in vitro and that potently inhibits angiogenesis and tumor growth in vivo [23,24]. Systemic therapy with recombinant endostatin results in tumor regression via a complete inhibition of angiogenesis [25]. Endostatin appears to be a highly specific inhibitor of endothelial cell proliferation and/or migration [26]. Reports have indicated that subconjunctive injection of the pBlast-hEndostatin could significantly inhibit CNV [6]. June et al. [27] injected the recombinant endostatin–adeno-associated virus (AAV) subconjunctivally and inhibited mouse CNV. Nicosia et al. [28] indicated that *RGD* peptide could inhibit endothelial cells’ proliferation and migration. Hill et al. [29] reported that a tripeptide *RGD* inhibited both angiogenesis and tumor growth. Furthermore, Koh et al. [30] and Yohei et al. [31] demonstrated that the *RGD* motif was a potential recognition sequence for binding integrins and inhibits endothelial cells’ adhesion and proliferation. Saiki et al. [32] reported that the polymerization of the Arg-Gly-Asp (*RGD*) core sequence was better able to augment inhibition of cell adhesion and proliferation than use of a monovalent unit of *RGD* peptide. Ren et al. [33] have modified endostatin and have demonstrated that the change augments its antitumor activity, including tumor cell proliferation and migration, and angiogenesis.

Therefore, this study cloned the endostatin gene and changed the *RGIRGAD* sequence into *RGDRGD*, using the site-directed mutagenesis method, and constructed a PCI mammalian expression vector. The study followed a rabbit CNV model, following subconjunctival injection of the endostatin and the *RGDRGD*-endostatin gene mixed with nonliposomal lipid. We investigated native endostatin and modified endostatin containing an *RGDRGD* motif; both significantly inhibited rabbit CNV in vivo, but the construct with the *RGDRGD* motif was more effective. We found significantly less vascular area and fewer blood vessels in *RGDRGD*-endostatin-treated rabbit eyes than in the other groups. Furthermore, we observed low-concentration expression of VEGF in the aqueous humor. Immunohistochemical analysis suggested a very low expression of CD31 in the *RGDRGD*-endostatin group. The results indicate that modified *RGDRGD*-endostatin gene delivery may be useful as an angiogenic inhibitor for the control of CNV.

## Methods

### Animals

Forty-two inbred adult (female) New Zealand white rabbits weighing 2.0–2.5 kg each were obtained from Harbin Medical Hospital Experimental Animal Center, Harbin, P.R. China. Prior approval of the experimental protocol was obtained from the Harbin Medical University School Research Institutional Animal Care and Use Committee. All protocols and the treatment of animals were in accordance with the Association for Research in Vision and Ophthalmology (ARVO) Statement for the Use of Animals in Ophthalmic and Vision Research.

### Rabbit model of CNV

General anesthesia was induced with an intramuscular injection of 50 mg/kg ketamine hydrochloride and 10 mg/kg xylazine. Central corneal alkali wound was produced in both eyes of 40 rabbits by applying a 5-mm round filter paper, soaked in 1 N NaOH, for 60 s [34]. On the first day after saturation, endostatin and *RGDRGD*-endostatin and vector was injected into the superior bulbar conjunctiva. Twenty rabbits received subconjunctival injection of 5 mg (0.4 ml) of endostatin into the right eye and vector into the left eye. Twenty rabbits received the injection of *RGDRGD*-endostatin into the right eye and did not receive any injection in the left eye (experimental controls). The PCI vector, PCI-endostatin plasmid, or PCI-*RGDRGD*-endostatin plasmid (5 µg) was mixed with 3 µl of FuGENE 6 (Roche, Indianapolis, IN) in which 400 µl of serum-free medium was added, and injected subconjunctivally, twice a week. Four eyes of two rabbits received neither treatment nor alkaline burn and served as absolute normal controls. Then, gentamicin ointment was applied to all eyes once per day.

### Site-directed mutagenesis and construction of plasmids

We have successfully constructed pMD18-T-endostatin (Takara, Tokyo, Japan) and conserved the plasmid of the pMD18-T-endostatin in our laboratory. The following primers were used to amplify the *RGDRGD*-endostatin gene by the rapid, site-directed mutagenesis technique, respectively: sense, 5′-CGG GGC GAC CGC GGG GAC TTC CAG T-3′; antisense, 5′-ACT GGA AGT CCC CGG CGG TCG CCC CG-3′ (Yingjun Biologic Engineering Technology, Shanghai, China). The site-directed mutagenesis PCR reaction consisted of 18 cycles of 5 min at 95 °C, 60 s at 94 °C, 45 s at 55 °C, 60 s at 72 °C, and 10 min at 72 °C. The products of PCR were precipitated by ethanol precipitation and DpnI enzyme digestion (New England Biolabs, Ipswich, MA). The products of digestion were transformed and the positive clone was verified.

PCI mammalian expression vector (Promega, Madison, WI), which has a CMV(cytomegalovirsu) promoter, was used for endostatin expression. The native and modified endostatin genes were subcloned into the XbaI-SalI sites of the PCI vector (Promega), which was then introduced into *E. coli* DH5α (Invitrogen, Carlsbad, CA) and ampicillin-resistant clones were selected. The amplified sequence was further confirmed with automated sequencing by Sangon Technology (Shanghai Sangon Biologic Engineering Technology, Shanghai, China). Plasmids were purified using the QIAprep Spin Miniprep Kit from Qiagen (Valencia, CA).

### Examination of exogenous endostatin gene expression in the transfected cornea

In a separate experiment to assess the exogenous protein expression and the duration of exogenous endostatin and *RGDRGD*-endostatin mRNA expression in the transfected rabbit cornea, total RNAs were prepared from rabbit cornea tissues at 3, 5, and 7 days after endostatin, *RGDRGD*-endostatin, and vector gene injection into the right and left eyes, respectively (n=3 for each time period). Total RNA (400 µg) was reverse-transcribed with random primers and MMLV by the RT–PCR kit (Promega). PCR amplification of cDNA was performed using RT–PCR and the primer sets were sense 5′-ATA GCC AGC GAA TTC ATG CAC AGC CAC-3′ and antisense 5′-ATT GTC GAC CTA CTT GGA GGC-3′. The RT–PCR reaction consisted of 30 cycles of 5 min at 95 °C, 50 s at 94 °C, 45 s at 55 °C, 60 s at 72 °C, and 10 min at 72 °C. A specific PCR primer for rabbit glyceraldehydes, 3-phosphate dehydro-genase (*GAPDH*) was designed based on the cDNA sequences (465 bp): sense,5′-GCT CCT GGT CAC CAG GGC TGC TT-3′; antisense, 5′-TGC CGA AGT GGT CGT GGA TGA CCT-3′(Yingjun Biologic Engineering Technology, Shanghai,China). The PCR products were separated by electrophoresis on a 1.0% agarose gel containing ethidium bromide.

### Evaluation

Completion of the clinical evaluations of CNV was as previously described [35]. At 7 and 14 days, rabbits in four randomized groups were examined, with 10 rabbits in each group. Two investigators examined CNV daily by a slit-lamp biomicroscope and monitored angiogenic responses. The area of CNV was determined by measuring the vessel length (L) from the limbus and the number of clock hours (C) of limbus involved [36-39]. Only the uniform contiguous band of neovascularization adjacent to the suture was measured. A formula was used to determine the area [37]: C/12×3.1416×[r^2^-(r-L)^2^], with C representing the clock hours of CNV; L representing the length of CNV, the ends of which were conjoined with each other; and r representing the half diameter of rabbit cornea (6 mm).

### Analysis of VEGF in aqueous humor

At 7 and 14 days, rabbits in the four randomized groups were anesthetized, and 50 μl aqueous humor was collected from each for determination of VEGF. The level of VEGF was measured in the aqueous humor with an enzyme-linked immunosorbent assay kit for human VEGF (R&D Systems, Minneapolis, MN) [40]. The VEGF kit detected two of the four VEGF isoforms (VEGF_121_ and VEGF_165_). The levels of these factors in the aqueous humor were within the detection ranges, with the minimum detectable concentration being 15.6 pg/ml for VEGF (intra-assay coefficient of variation [CV]: 5.5%; inter-assay CV: 6.9%) [41].

### Histology and immunohistochemistry

#### Light Microscopy

Corneal buttons were collected from the four groups and the absolute normal controls at the time of maximal neovascularization (day 14) after alkaline burn for the control and empty vector groups. The animals were anesthetized with ketamine hydrochloride and xylazine, and the common carotid artery was exposed. With a catheter introduced into the common carotid artery, PBS (Phosphate buffer saline) was flushed, followed by 4% paraformaldehyde. The eyes were enucleated and immediately placed into the same fixatives. After fixation, the corneas were excised and immersed in 4% paraformaldehyde fixative overnight at 4 °C. The tissue blocks were washed, dehydrated, and embedded in paraffin; 5 μm thin paraffin sections were stained with hematoxylin and eosin (H&E).

#### Immunohistology

Corneas were examined using immunohistochemical methods for four rabbits from each group. Serial olefin sections (5 μm) of each eye were prepared and immunohistochemical methods analyzed the expression of VEGF (Santa Cruz Biotechnology, Santa Cruz, CA). To visualize the vascular endothelial cells and determine the degree of induced angiogenesis, sections were stained with anti-CD31 (Abcam, Cambridge,MA). Four representative sections obtained from each cornea from the control, PCI emptyh vector-injected, PCI-endostatin, and PCI-*RGDRGD*-endostatin groups were analyzed with a microscope (Olympus, Tokyo, Japan) as described [42]. To visualize the inflammatory cells, sections of the retina, liver, and kidney were stained with anti-CD38 antibody (Abcam) on day 90. The immunohistochemistry process was performed according to the avidin-biotin complex (ABC) method.

Thin paraffin sections (5 μm) were mounted on silanized slides, dried overnight, and deparaffinized with descending concentrations of ethanol and xylene. The slides were placed into citrate buffer (pH=6) in a boiling pot for 5 min. After cooling for 30 min, the specimens were incubated with 5% normal bovine serum for 30 min, followed by incubation with the primary antibody in humidified chambers for 2 h at room temperature. The primary antibodies used were anti-VEGF antibody (1:400), anti-CD31 antibody (1:400), and anti-CD38 (1:400) plus 1% normal rabbit serum, respectively. For detection, the 3-step ABC was used. The secondary biotinylated antibody was a goat anti-mouse immunoglobulin G (Abcam) antibody diluted 1:200. Finally, the slides were incubated with streptavidin and alkaline phosphatase. The phosphatase activity was visualized by fast red solution (Sigma, St. Louis, MO), yielding a reddish staining. The sections were slightly counterstained with hematoxylin. Negative controls were without primary antibody.

The images were captured with a Spot digital camera (Media Cybernetics, Silver Springs, MD), and morphometric analyses were performed using Image-Pro Plus software (Media Cybernetics). The whole field at 200× magnification was examined on each cross-section, and the sum and mean of the vascular area of each vessel and the number of vessels per section were determined.

### Statistical analysis

All statistical analyses were performed using SPSS 15.0 software (SPSS, Chicago, IL). The statistical significance of differences between the control, vector, endostatin, and *RGDRGD*-endostatin gene-treated groups and between the endostatin and *RGDRGD*-endostatin groups were determined using ANOVA (ANOVA) and the unpaired Students’ *t*-tests. Differences were accepted as significant at p<0.05.

## Results

### Construction of vectors containing native and modified endostatin genes

**Figure 1:**
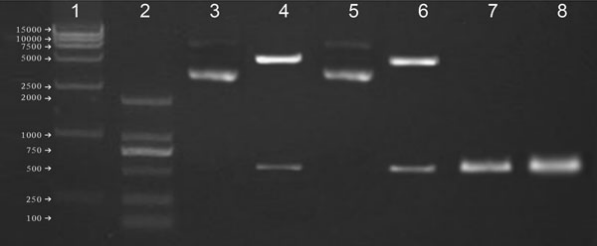
Construction of vectors containing human native and modified endostatin genes. Identification of PCI-endostatin and pCI-*RGDRGD*-endostatin. Lane 1: 15,000 marker; Lane 2: 2,000 marker; Lane 3: PCI-endostatin; Lane 4: PCI-endostatin (SacI +SalI); Lane 5: PCI-*RGDRGD*-endostatin; Lane 6: PCI-*RGDRGD*-endostatin (SacI +SalI); Lane 7: PCR product of native endostatin gene; Lane 8: PCR product of modified *RGDRGD*-endostatin gene.

**Figure 2:**
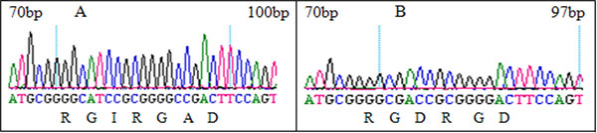
The sequence analysis of the endostatin and modified *RGDRGD*-endostatin genes. **A**: The sequence analysis of the endostatin gene in the range of 70 to 100 bp. **B**: The sequence analysis of the modified *RGDRGD*-endostatin gene in the range of 70 to 97 bp. The sequence analysis indicated that a modified endostatin gene was obtained by delete alanine ala (A) and isoleucine ile (I) changed to asparticacid asp (D), and that the *RGDRGD* motif was successfully constructed.

To study the biologic activity of the recombinant modified human endostatin gene containing an *RGDRGD* motif, we modified the human native endostatin gene by a rapid site-directed mutagenesis technique, constructed mammalian expression vectors, and confirmed by sequencing (Figure 1 and Figure 2). Compared with human endostatin genes in the GenBank (NM_130445), the genes used in this study had two mutant sites: the 273 base, mutated from G→T, and the 530 base, mutated from A→C. They were silent mutations and the coded amino acid was unchanged.

### Endostatin expression in rabbit cornea after local gene therapy

**Figure 3:**
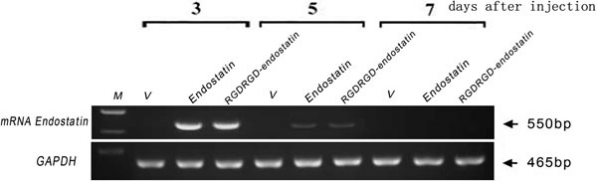
RT–PCR analyses assessed the duration of endostatin or *RGDRGD*-endostatin mRNA expression in the rabbit cornea. The expression of the human endostatin or *RGDRGD*-endostatin gene (550 bp) was observed until 5 days after single subconjunctival gene injection, while there was no expression of after vector (V) injection. The results of rabbit *GAPDH* (465 bp) confirmed the relative amounts and fidelity of the total RNA samples.

To confirm the efficiency of local gene transfer in the CNV model, the expression of human endostatin and *RGDRGD*-endostatin mRNA in the rabbit cornea was observed until 7 days after subconjunctival gene injection (Figure 3). Thus, we injected the endostatin or *RGDRGD*-endostatin plasmid or vector gene twice a week to maintain the duration of the transgene expression.

### Biomicroscopic examination and scoring of neovascularization

**Figure 4:**
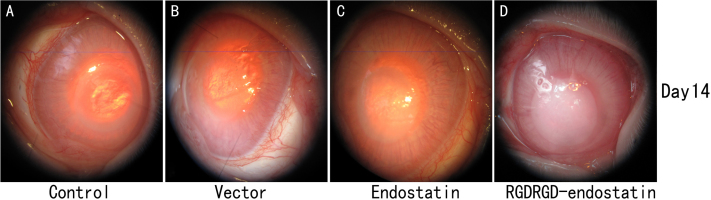
Biomicroscopic findings in corneas after subconjunctival injection of endostatin-*RGDRGD* or endostatin or vector, and in untreated control corneas. Representative biomicroscopic findings of rabbit corneas are shown. The corneas were examined at 1, 7, and 14 days after injection. The endostatin-*RGDRGD*-treated eyes showed significantly less neovascular growth than endostatin-treated eyes or vector-treated eyes or untreated eyes at day 7, and peaked on day 14.

The rabbit eyes were examined under a surgical microscope daily. CNV increased gradually, peaked on days 12–14, and degenerated on day 16 after suture induction. Therefore, we chose days 7 and 14 as time points to determine the number and length of vessels and the area of neovascularization. The corneal opacity and angiogenesis gradually increased in three groups. However, there was less angiogenesis observed in the *RGDRGD*-endostatin-treated group than in the other groups (Figure 4).

**Figure 5:**
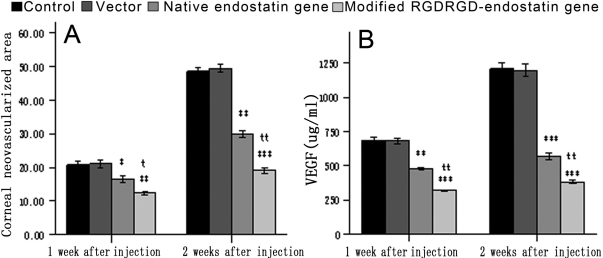
The inhibitory effect of modified *RGDRGD*-endostatin gene on the area of CNV and the expression VEGF in aqueous humor. **A**: The inhibitory effect of modified *RGDRGD*-endostatin gene on the area of CNV. The area of CNV was calculated by the number of newly developed vessels multiplied by the length of the vessels from the limbus, 7 and 14 days after the alkaline burn. **B**: The concentration of VEGF in aqueous humor (n=10, M±SD). The modified *RGDRGD*-endostatin gene has a significantly different effect compared with the native gene at 7 and 14 days. Data are expressed as a mean±SD, as well as at other days. ***p<0.001; **p<0.01; *p<0.05, when CNV with treated groups was compared with control group. ^t^p<0.05, ^tt^p<0.01, when CNV with the combination of *RGDRGD*-endostatin was compared with CNV treated with native endostatin.

The mean areas of CNV were 21.08±4.21, 20.24±5.36, 16.24±2.16, and 12.56±1.12 at day 7 and 48.47±3.08, 49.18±4.29, 30.86±3.46, and 19.46±2.45 at day 14 for the control, empty vector, endostatin, and *RGDRGD*-endostatin groups, respectively. Analysis of the CNV area indicated that local application of *RGDRGD*-endostatin resulted in a significant suppression of CNV (Figure 5A).

### VEGF detected in aqueous humor

The study first examined whether native endostatin and modified endostatin with *RGDRGD* motif genes would inhibit the expression of VEGF detected in aqueous humor. The concentration of VEGF in aqueous humor was detected for 10 rabbits from each group at 7 and 14 days. Analysis of the concentration of VEGF in aqueous humor indicated that local application of *RGDRGD*-endostatin resulted in a significant suppression of the expression of VEGF (Figure 5B).

The concentrations of VEGF in the absolute normal control group were 3.6, 3.8, 3.5, and 3.4. The mean concentrations of VEGF in the aqueous humor were 681±40, 689±54, 486±35, and 319±21 at day 7 and 1,221±116, 1,214±121, 566±47, and 385±31 at day 14 for the control, empty vector, endostatin, and *RGDRGD*-endostatin groups, respectively (Figure 5B).

### Histology analysis and expression

**Figure 6:**
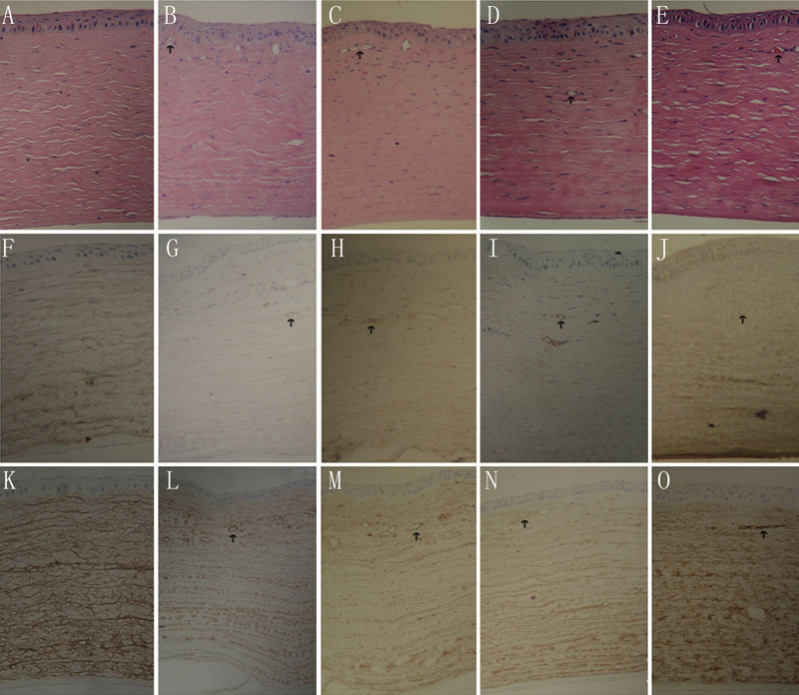
Histologic profiles of the corneal buttons . Corneal buttons were collected on day 14 (**A**-**E**). Corneal button sections of normal (**A**),control (**B**), Vector (**C**), endostatin (**D**), and *RGDRGD*-endostatin (**E**) groups were stained with hematoxylin and eosin. Expression of VEGF in the corneal buttons was detected by immunohistochemistry (**F**-**J**). High levels of VEGF were detected in the control and vector groups, respectively (**G**-**H**); by contrast, only minute levels were detected in the endostatin and *RGDRGD*-endostatin gene groups (**I**-**J**), respectively . Light micrographs of CD31-stained corneas in an untreated eye after corneal denudation and the effects of RGDRGD-endostatin gene delivery on experimental corneal angiogenesis on day 14. Representative light micrographs of CD31-stained corneas given normal, control,vector,endostatin,or the RGDRGD-endostatin expression cassette are shown (each group; n=3; **K**-**O**).The absolute normal control group showed no neovascularization, the experimental normal and vector groups showed neovascular formation (arrowheads), the endostatin group showed less neovascular formation,while the RGDRGD-endostatin group showed significantly less vessels and smaller areas of neovascularization compared with endostatin-treated corneas.

To examine the change of CNV in the histology, corneal button sectons were stained with hematoxylin and eosin. Figure 6A showed a normal corneal button section. No CNV were detected. Many CNV were detected in the control and empty vector groups (Figure 6B,C). There were red blood cells in the lumen. In the endostatin and RGDRGD-endostatin groups, less CNV were detected (Figure 6D,H).

To examine the change in the histology and the expressions of VEGF and CD31, pathology and immunohistochemistry were performed. In the control, empty vector,endostatin,and RGDRGD-endostatin, groups, VEGF were detected in the cornea of all allografts (Figure 6G-J). Furthermore, we used CD31 staining as a molecular method to detect neovascularization. Immunohistochemical studies showed significant differences among these groups in the expression of CD31. In the control and empty vector groups, high levels of CD31 were detected in the corneal of all allografts (Figure 6L,M). In the endostatin group, a lower level of CD31 was expressed (Figure 6N). By contrast, its expression levels were very low but detectable in the *RGDRGD*-endostatin group (Figure 6O). These findings suggest that *RGDRGD*-endostatin augmented the suppression of the expression of CD31 and neovascularization development. The *RGDRGD*-endostatin-treated eyes showed significantly less neovascular area and fewer vessels than untreated eyes, vector-treated eyes, or endostatin-treated eyes. Histological analysis revealed the results of neovascular area and number of vessels in *RGDRGD*-endostatin-treated, endostatin-treated, vector-treated, and untreated control eyes (Table 1).

**Table 1:** The mean data for the neovascular area of each vessel and the number of vessels in sectioned corneas of gene-treated groups.

	**Groups(area/vessel number)**			
**Days**	**Control**	**Vecto- transfected**	**Endostatin-transfected**	**RGDRGD-transfected**
14 day after injection	874.2±64.2/8.1±0.5	826±58.9/12.7±0.7	498.3±49.2/3.8±0.3	237.2±73.1**/1.6±0.4*

Values are mean±SD (n=3).The vascular area (mm^2^) of each vessel and number of vessels in three representative corneal sections of each rabbit were analyzed by histological examination and morphometric analyses as described in Methods. Asterisks indicate significant differences between the corresponding *RGDRGD*-endostatin and endostatin-transfected groups (*p<0.05; **p<0.01).

To examine whether there were inflammatory cells in the histopathology of the retinal, hepatic, and renal tissue sections, the sections were stained with anti-CD38 antibody. Positive controls showed CD38+ inflammatory cells adjacent to biliary cirrhosis in hepatic tissue sections. A comparison of staining showed a significant difference in the expression of CD38. In the retinal, hepatic, and renal tissue sections, the organization is normal, no inflammatory cell infiltration.

**Figure 7:**
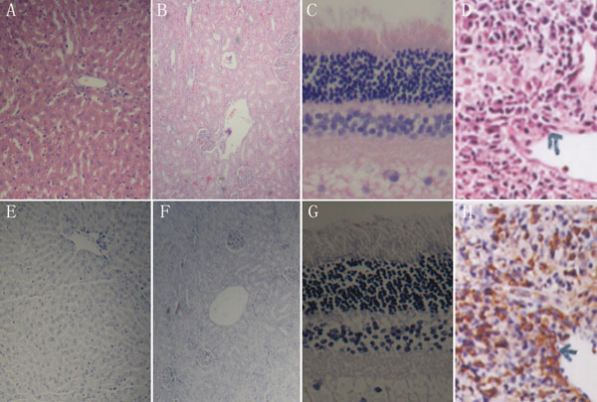
Histological profiles of retinal, hepatic, and renal tissue sections at 90 days. Retinal, hepatic, and renal tissue sections were normal, and no inflammatory cell infiltration was observed (**A**-**C**). Immunohistochemical studies indicated that CD38+ cells were not detected in the retinal, hepatic, and renal tissue sections (**E**-**G**), respectively. In positive controls, the expression level of CD38 rised significantly. CD38+ cells (arrowheads) were detected in the hepatic tissue sections (**D** and **H**), respectively.

Immunohistochemical studies showed that the levels of CD38 were not detected in the retinal, hepatic, and renal tissue sections (Figure 7).

## Discussion

The cornea is a transparent anterior ocular tissue and protects the eyeball from outside stimuli [1]. The avascular nature of the cornea is crucial for maintaining its immune-privileged status. CNV, which occurs in many pathologic states of the cornea, reduces visual acuity [1]. Angiogenesis is a complex process that includes the activation, proliferation, and migration of endothelial cells; the disruption of the vascular basal membrane; and the formation of vascular tubes and networks, and it connects new and preexisting vascular networks [43]. Various angiogenic factors mediate CNV, including VEGF, the basic fibroblast growth factor, matrix metalloproteinase, and insulin-like growth factor-1 [1]. VEGF appears to be the most prominent angiogenic factor. Inhibition of VEGF activity is highly effective for suppressing angiogenesis.

Previous studies show that human endostatin inhibits tumor neovascular growth by suppressing VEGF expression [44,45]. Human endostatin, a 20-kDa COOH-terminal fragment derived from type XVIII collagen, is a potent angiogenesis inhibitor and an antitumor factor [46]. The COOH-terminus 315 or 313 residues of collagen type XVIII are noncollagenous and form the NCI domain. Endostatin, specifically, inhibits vascular endothelial cell proliferation and is recognized as one of the most potent antiangiogenesis agents at present. This factor also has the benefits of nontoxic effects or drug resistance after long-term use. It is an ideal biologic antiangiogenesis agent. Because the N-terminal of endostatin has an internal *RGIRGAD* sequence at position 25–31, the *RGD* motif is a potential recognition sequence for binding integrins. Whereas this study successfully changed the *RGIRGAD* sequence into an *RGDRGD* sequence, Ren [33] identified that the ploy *RGD* structure in endostatin could augment biologic activity. An alternative explanation, given our observations in the rabbit CNV model, could be that modified *RGDRGD*-endostatin exhibited greater inhibition of CNV in the rabbit alkaline burn model. Meanwhile, the inhibitory effects of modified *RGDRGD*-endostatin on rabbit CNV were greater than the effects of native endostatin.

The cornea is readily accessible for gene therapy in the laboratory and in the clinic. The method of corneal injection of a plasmid containing the cDNA is safe, effective, titratable, and easily monitored [1,47]. FuGENE 6 Transfection Reagent showed very low cytotoxicity and delivered high transfection efficiency. The FuGENE 6 used in this study is nontoxic, feasible, efficient, and safe for the cornea in the gene transferring [1,48,49]. Yoon et al. [1] reported that the subconjunctival injection of pEGFP-BAI1-ECR plasmid or pEGFP vector produced green fluorescence in the corneal stroma of both groups [1]. In this study, the expression of the human endostatin or *RGDRGD*-endostatin gene was observed at 3 days; the expression was decreased at 5 days, and at 7 days there was much less expression after a single subconjunctival gene injection, while there was no expression after vector injection. Thus, the endostatin, *RGDRGD*-endostatin plasmids, or vector gene were injected twice a week to maintain the duration of transgene expression.

In this study, we successfully obtained modified endostatin genes by rapid site-directed mutagenesis and constructed a PCI-endostatin-*RGDRGD* mammalian expression vector. The lipid delivery of the *RGDRGD*-endostatin gene was effective at inhibiting neovascularization, which is consistent with the Ren et al. report [33] that ploy *RGD* motif augments the biologic activity of endostatin; this is also consistent with Saiki et al. [32].

The histology assay indicated that the *RGDRGD*-endostatin-treated group had fewer vessels and smaller areas of neovascularization compared with three groups by labeling vascular endothelial cells by CD31. Immunohistochemical studies suggested that endostatin and modified *RGDRGD*-endostatin could inhibit expression of VEGF and neovascularization development, compared with the control and vector groups, which is consistent with the Hosseini et al. [14] report. Under the light microscope, retinal, hepatic, and renal tissue sections were normal, and no inflammatory cell infiltration was observed at 90 days. Immunohistochemical studies indicated that three tissue sections were normal, and no plasmocyte infiltration was observed at 90 days. These findings suggest that *RGDRGD*-endostatin is a strong and effective antiangiogenic agent.

This study successfully obtained a modified endostatin gene and constructed mammalian expression vectors by rapid site-directed mutagenesis. The results show that modified endostatin genes containing *RGDRGD* motif could augment the inhibition of the expression of VEGF, significantly reducing levels of VEGF, and that modified endostatin genes containing *RGDRGD* motif could inhibit the development of neovascularization more effectively than native endostatin. These results indicate that modified *RGDRGD*-endostatin gene delivery effectively reduces experimental CNV.
